# Has Adolescent Childbearing Been Eclipsed by Nonmarital Childbearing?

**DOI:** 10.3390/soc5040734

**Published:** 2015-10-28

**Authors:** Anne Martin, Jeanne Brooks-Gunn

**Affiliations:** National Center for Children and Families, Teachers College, Columbia University, 525 W. 120th Street, Box 39, New York, NY 10027, USA

**Keywords:** adolescent childbearing, nonmarital childbearing

## Abstract

Adolescent childbearing has received decreasing attention from academics and policymakers in recent years, which may in part reflect the decline in its incidence. Another reason may be its uncoupling from nonmarital childbearing. Adolescent childbearing became problematized only when it began occurring predominantly outside marriage. In recent decades, there have been historic rises in the rate of nonmarital childbearing, and importantly, the rise has been steeper among older mothers than among adolescent mothers. Today, two out of five births are to unmarried women, and the majority of these are to adults, not adolescents. Nonmarital childbearing is in and of itself associated with lower income and poorer maternal and child outcomes. However, unmarried adolescent mothers might face more difficulties than unmarried adult mothers due to their developmental status, education, living arrangements, and long-term prospects for work. If this is true, then the focus on adolescent mothers ought to continue. We suggest several facets of adolescent motherhood deserving of further study, and recommend that future research use unmarried mothers in their early 20s as a realistic comparison group.

It is our impression that the amount of attention paid by academics, policymakers, and the public to adolescent childbearing as a social problem in the U.S. has markedly declined since the 1980s. The issue became a national policy priority in 1984, when a panel of experts on teenage pregnancy and childbearing was convened by the National Research Council, and funded by a host of private foundations. Three years later, the panel issued its landmark report, *Risking the Future: Adolescent Sexuality, Pregnancy, and Childbearing* [1], which addressed the causes and consequences of these phenomena. This report became an authoritative source for at least a decade. A quick search in Google Scholar reveals that it has been cited over 91,000 times. Teenage pregnancy and childbearing remained a central policy focus in the 1990s, as their rates peaked in 1990 and 1991, respectively [2]. In 1996, the National Campaign to Prevent Teen Pregnancy was founded, declaring its mission to reduce the adolescent pregnancy rate in the U.S. by one-third over the next decade (thenationalcampaign.org/about/history).

Over the 2000s, according to our admittedly impressionistic observations, scholarly and public interest in adolescent childbearing diminished. One very good reason for this may be that the birth rate among adolescents has been on the decline since 1991 [3]. For example, there were 62 births for every 1000 female 15–19-year-olds in 1980. This rate fell to 48 births per 1000 by the year 2000, and as of 2013 it was 27 births per 1000 [3]. Adolescent pregnancy rates have also fallen since 1991 [4]. Most of this decline appears to be attributable to improved contraceptive use [5]. Perhaps tellingly, the National Campaign to Prevent Teen Pregnancy, having reached its goal, broadened its mission to include unplanned pregnancies for women of all ages.

We propose here that in addition to their drop in rates, another factor that may have contributed to adolescent pregnancy and childbearing’s retreat from public view was a related demographic trend unfolding over the same period: the increase in nonmarital childbearing. One of the primary concerns raised about adolescent childbearers in the 1970s and 1980s was their overwhelming likelihood of being unmarried. Part of this concern among the public and some advocates was, no doubt, driven by misgivings about the morality of nonmarital childbearing. However, the concerns of scholars and public health practitioners rested on the troubling observation that the children of unmarried mothers fared worse than those of married mothers.

This issue gained new recognition as a social problem with the publication in 1994 of *Growing Up with a Single Parent* [6], in which McLanahan and Sandefeur reported that adolescents who had grown up in single-parent families had poorer academic performance, lower college enrollment, and higher birth rates than those in two-parent families. Additional evidence accrued in the 1990s showing that poorer academic and behavioral outcomes were found among the younger children of single mothers as well (for a review, see Sigle-Rushton and McLanahan [7]). Our current knowledge base indicates that children born outside marriage have lower academic scores and higher behavior problem scores than children born within marriage, although associations are moderated by biological father involvement and the presence of other father figures over the course of childhood (for a review, see Waldfogel, Craigie, and Brooks-Gunn [8]). In addition, it is clear that women who give birth outside marriage have lower educational attainment and income than women whose births occur within marriage (for a review, see McLanahan and Percheski [9]).

It should be acknowledged that the link between marriage and better maternal and child outcomes is not thought to be fully or even predominantly causal. One study found that the children of married and unmarried parents scored similarly on achievement once controls were in place for self-selection into marriage among women and men with more education, men without criminal backgrounds, and women with higher achievement [10]. However, the children of married parents had better behavior scores even adjusting for parental self-selection into marriage. Nevertheless, it is clear that compared to married mothers, unmarried mothers receive less financial and instrumental support from their children’s biological fathers, have lower-quality co-parenting relationships with those fathers, and are more likely to be stressed and depressed [8].

To be sure, the disadvantage of single motherhood is not the sole reason adolescent childbearing has been viewed as problematic by scholars and practitioners. A greater proportion of teenagers’ births than older women’s births are unintended [11]. Teenage mothers are less likely than older mothers to obtain prenatal care [12]. The cost of the medical and social services associated with adolescent childbearing and parenting is often borne by taxpayers because adolescent mothers are typically unable to pay [13]. Teenage mothers’ youth and immaturity may inhibit optimal parenting behaviors. Some research indicates that compared to older mothers, adolescent mothers are more punitive, less sensitive and less stimulating with their young children [14–16].

Still, the problematization of adolescent childbearing has always highlighted its occurrence outside marriage. In the 1950s and 1960s, the birth rates for adolescents were far higher than they are today, but they were not viewed as problematic because the vast majority of those births occurred within marriage [3]. Although the birth rate for adolescents declined between the 1950s and the 1970s, the proportion of those births that were nonmarital grew [3]. Thus, by 1980, approximately half of adolescent births occurred outside marriage, and by 1990, 67% did so [3].

At the time *Risking the Future* was released, adolescent and nonmarital childbearing overlapped significantly. That is, not only were most adolescent births nonmarital, but additionally, a disproportionate number of nonmarital births were to adolescents. However, between 1980 and the present, a remarkable demographic and social transition unfolded. The incidence of nonmarital childbearing skyrocketed. The nonmarital birth rate (number of births per 1000 unmarried women aged 15–44) in 1980 was 29.4; by 2011, it was 46.0 [17]. Only 18% of births in 1980 were to unmarried women, but by 2000, that figure was 33%, and by 2010, it was 41% [17]. The current average masks much higher rates among particular subgroups of women. Among Hispanics in 2010, 53% of all births were nonmarital, and among blacks, fully 72% of all births were nonmarital [17].

Notably, the rise in nonmarital births has been steeper for mothers in their 20s and older than it has been for adolescents, as illustrated in Figure 1. Between 1970 and 2011, the increase in the birth rate among unmarried women aged 15–19 was 27%, compared to 74% for 20–24 year olds, 83% for 25–29 year olds, 107% for 30–34 year olds, 120% for 35–39 year olds, and 134% for 40–44 year olds ([17] Table 16). While it is true that adolescents remain more likely than older women to give birth outside marriage, it is no longer the case that nonmarital births characteristically occur to adolescents. In 1970, half of all births to unmarried women were to adolescents, but as of 2007, only one-quarter were [18]. By comparison, 42% of unmarried births in 1970 were to women in their 20s, compared to 60% (the majority) in 2007 [18].

These trends have radically transformed the relationship between adolescent and nonmarital childbearing. Indeed, over time, as teenage childbearing has receded from public and academic attention, nonmarital childbearing has assumed growing visibility. In 1996, the welfare reform legislation (the Personal Responsibility and Work Opportunity Reconciliation Act, or PRWORA) included, as one of its aims, the promotion of marriage among low-income couples. The initiation of the Fragile Families and Child Wellbeing Study in 1998–2000, sponsored by the National Institutes of Health and several private donors, signaled a new era in the study of nonmarital childbearing. This study recruited nearly 5000 newborns in 20 large U.S. cities and over-represented nonmarital births by design [19]. The study is now following subjects as they reach their 15th birthday, and has been the source for hundreds of publications addressing the parenting, family formation, and fertility behaviors of unmarried parents, as well as the cognitive and socioemotional development of their offspring.

Thanks in large part to this study, it is now abundantly clear that nonmarital childbearing occurs disproportionately among the most socioeconomically disadvantaged women, but that pre-existing disadvantage does not fully account for deficits in family income and maternal mental health later in life [20,21]. Nevertheless, it does not appear wise from a policy perspective to encourage marriage among low-income women in our current economy because the bulk of men in their marriage pool are unappealing due to their low earnings. Moreover, unmarried men with histories of substance use, criminality, and infidelity may end up being drains on the household [20,22,23]. With respect to adolescents in particular, there is no evidence to suggest that promoting marriage would be advantageous. Although few scholars have looked at marriage among adolescent mothers, likely because of its infrequency, Mollborn [24] found that married teenage mothers had lower educational attainment than other teenage mothers, perhaps because they were burdened by caregiving duties.

The seismic shift in nonmarital childbearing among non-adolescent women of reproductive age has important implications for scholars struggling to understand and quantify the unique disadvantage conferred by adolescent childbearing to both mothers and offspring. We propose that serious thought be given to the question of who should constitute the appropriate comparison group for teenage mothers. In the 1950s and 1960s, when adolescent childbearing within marriage was normative, the primary counterfactual condition for adolescents who became mothers—who were by and large not married—was having a baby during adolescence within the context of marriage. In the 1970s and 1980s, once childbearing during adolescence was no longer normative, the primary counterfactual condition for adolescents who became mothers—who were still by and large not married—was delayed childbirth until their 20s, when their chances of marriage would improve. Currently, the primary counterfactual condition for adolescents who become mothers—who remain by and large not married—is delayed childbirth until their early 20s, when they are likely to remain unmarried. We specify the early 20s rather than the late 20s as a comparison group because it is not realistic to expect public health and welfare programs to convince adolescent women who are apt to become mothers to defer childbearing for more than approximately five years.

There is, therefore, a need for research that delineates the costs and consequences of nonmarital childbearing during adolescence compared to the costs and consequences of nonmarital childbearing during the early 20s. Further, this research should account for the advantages of childbearing within each life stage, such as better grandmaternal health and thus greater odds of receiving help with childrearing among African Americans during adolescence [25]. The reward of conducting research with a clearly and thoughtfully selected comparison group is that it should help us estimate more realistic projections of the gains to be yielded by programs and policies designed to prevent adolescent childbearing, because the comparison group should look like what the group targeted by the program would look like if the program were to succeed. Another advantage of a comparison between adolescent mothers and mothers in their early 20s is that it may allow us to identify features of motherhood in the latter group that point to previously undetected maturational processes occurring in emerging adulthood.

We see at least three areas relevant to the lives of adolescent mothers today that have a pressing need for more research. First, adolescent mothers are likely to live with their mothers for at least the first part of their child’s life [26], but past studies suggest that three-generation households can be problematic for families, particularly white families, among whom extended family living is considered non-normative [27]. It appears that coresident grandmothers provide child care and financial assistance to teenage mothers [26], resulting in mothers’ increased involvement in school and work, and by their late 20s, greater educational attainment [24]. But coresidence with the grandmother is also associated with mothers’ decreased involvement in parenting [28] and poorer parenting skills [29–32].

The coresidence of an adolescent mother’s own mother may reinforce her more in the role of daughter than mother [33]. Coresidence may also provoke greater mother-grandmother conflict [28], which in turn detracts from the mother’s parenting [34]. Individuation from parents is a normal developmental task of adolescence, but this process is thwarted when adolescent mothers rely on their mothers for key material and emotional assistance with raising their young child. It is a challenge for scholars and practitioners alike to envision strategies for helping adolescent mothers forge an independent identity from their mother while sharing caregiving duties for a young child. Yet the goal is a worthy one. Two small studies of urban adolescent mothers found that those with greater individuation from their mother had higher-quality parenting skills [35,36]. Efforts are needed to understand what kind of programs might support adolescent mothers who coreside with their own mothers that would maximize the young mother’s feelings of autonomy, while acknowledging her dependence on and indebtedness to her mother.

Second, we need to understand more about the biological fathers and social fathers of children mothered by adolescents. We already know that relationships between adolescent mothers and their baby’s biological father tend to be conflictual [37] and short-lived [38–40]. Past research shows that adolescent mothers are distressed by the uninvolvement of the biological father [41,42], but less is known about how they are affected by the involvement of new romantic partners as their child ages, particularly if a nonmarital union results in a new baby. The presence of new romantic partners is likely to be swift following an adolescent’s birth. One study of adolescent mothers in Baltimore found that half were in a new romantic relationship within two years of childbirth [43].

From a child development perspective, romantic partners who move in with an adolescent mother and her child are particularly worrisome because they are not likely to stay, and instability in family composition undermines optimal child development. However, the experience of multiple coresident father figures is becoming increasingly common. The 1990s and 2000s saw a spike in serial cohabitation, and this phenomenon is now particularly prevalent among women who give birth as adolescents [44]. This development does not bode well for the children of adolescent mothers. Multiple coresidential partner transitions are associated with lower academic test scores and more behavior problems in children as early as age five [8,45]. Thus, there is a pressing need for research on factors that promote stability in adolescent mothers’ relationships, and factors that buffer mothers and children from the effects of instability.

Third, further study is needed to investigate the child care arrangements secured by teenage mothers. Plentiful research documents the cognitive advantages conferred by high-quality center-based care and education during the first five years of life [46–48], but adolescent mothers tend to be low-income and may be unable to afford or find access to such care arrangements. Additionally, their home environments may be less stimulating and nurturing than older mothers’ owing to a lack of maturity and education. A recent national study found that adolescent mothers who were full-time caregivers for their child had poorer outcomes when the child was age four, than adolescent mothers who used child care [49]. Additionally, compared to adolescent mothers who were full-time caregivers, those using center-based child care were less likely to have a rapid repeat birth, and those either using center care or paying for home-based care had higher household incomes. Interestingly, the children of teenage mothers benefited more cognitively and behaviorally than the children of older mothers from the use of non-parental care. However, teenage mothers who were the exclusive caregivers came from more disadvantaged families than those who used non-parental care, suggesting that affordability may have been a primary consideration behind their care arrangement. There is thus a need for research exploring how adolescent mothers select their child care arrangements and whether they have access to the types of arrangements they prefer.

## Figures and Tables

**Figure 1:**
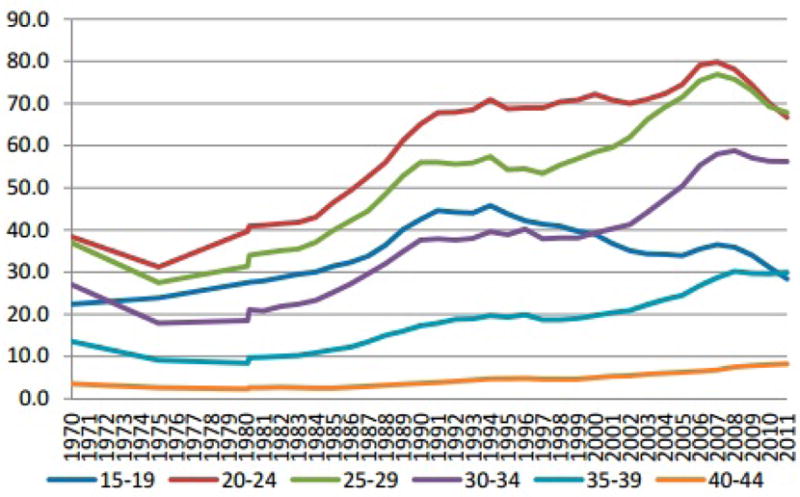
U.S. Birth Rates for Unmarried Women by Age of Mother (Source: Table 16 in [17]).
